# Supplementary material for: Multilevel theorizing in health communication: Integrating the Risk Perception Attitude (RPA) framework and the Theory of Normative Social Behavior (TNSB)
Source: PLoS One. 2022 Jul 22;17(7):e0271804. doi: 10.1371/journal.pone.0271804 (PMC9307156; doi:10.1371/journal.pone.0271804)
Supplement: Attachment — Submitted filename: PONE-D-21-35351_reviewer comment.docx [file pone.0271804.s001.docx]

This paper resorts the concept of efficacy to bring together normative factors at the social-level, specifically including descriptive norms, injunctive norms and collective norms, and risk perception at the individual-level, and analyzes data from the RANI project through hierarchical linear equations, and then examines the role of a series of predictors and efficacy on the intention to consume IFA. The main findings are the strength of the association between efficacy beliefs and intentions and the interaction effect involving efficacy beliefs.

The literature review of this paper is well prepared, the hypotheses are natural and reasonable, and the conclusions are generally credible. It is an honor to read this paper, but there are still a few suggestions before publication:

1. It is suggested that the authors supplement the hierarchical linear equations used in this paper at appropriate place, and further enrich the summary statistics on measures;
2. It is suggested that the authors provide further explanations for “*low reliability*” and “*exponentially iterative process*” in **Measures**.
3. As the authors mention the relationship between efficacy and behaviors in **Limitations**, it is also recommended that they explore the self-efficacy and response efficacy separately;
4. The dependent variable in this paper is the intention to consume IFA, not the actuals. If permitted, it is recommended that the authors continue to track the behavioral performance of participants to better supplement and evaluate the conclusions involved in this paper.
