# Supplementary material for: Multilevel theorizing in health communication: Integrating the Risk Perception Attitude (RPA) framework and the Theory of Normative Social Behavior (TNSB)
Source: PLoS One. 2022 Jul 22;17(7):e0271804. doi: 10.1371/journal.pone.0271804 (PMC9307156; doi:10.1371/journal.pone.0271804)
Supplement: Attachment — Submitted filename: Response to Reviewers.docx [file pone.0271804.s002.docx]

Dear Editor and Reviewers,

Thank you for taking the time to thoroughly review our manuscript PONE-D-21-35351

Multilevel theorizing in health communication: integrating the Risk Perception Attitude (RPA) framework and the Theory of Normative Social Behavior (TNSB). I am delighted to share our response to each editorial and reviewer comment below, indicated in bold blue font.

**Editors Comments:**

1. Please ensure that your manuscript meets PLOS ONE's style requirements, including those for file naming. The PLOS ONE style templates can be found at

<https://journals.plos.org/plosone/s/file?id=wjVg/PLOSOne_formatting_sample_main_body.pdf> and

<https://journals.plos.org/plosone/s/file?id=ba62/PLOSOne_formatting_sample_title_authors_affiliations.pdf>.

**Thank you for sharing these resources. We have now edited the manuscript so that it is in line with the journal’s style requirements. These edits include modifications to the title page, headings, figure file names and formats, and references.**

2. Please provide additional details regarding participant consent. In the ethics statement in the Methods and online submission information, please ensure that you have specified (1) whether consent was informed and (2) what type you obtained (for instance, written or verbal, and if verbal, how it was documented and witnessed). If your study included minors, state whether you obtained consent from parents or guardians. If the need for consent was waived by the ethics committee, please include this information.

If you are reporting a retrospective study of medical records or archived samples, please ensure that you have discussed whether all data were fully anonymized before you accessed them and/or whether the IRB or ethics committee waived the requirement for informed consent. If patients provided informed written consent to have data from their medical records used in research, please include this information.

**Thank you for identifying this important oversight. We have now included the relevant informed consent information in the manuscript (page 9).**

3. Please ensure you have included the registration number for the clinical trial referenced in the manuscript.

**We have now included the clinical trial registration number within our ethics statement (page 9).**

4. Thank you for stating the following in the Acknowledgments Section of your manuscript:

“This study was funded by the Bill and Melinda Gates Foundation (OPP1182519). The authors of this paper have no conflicts of interest to report.”

We note that you have provided funding information that is not currently declared in your Funding Statement. However, funding information should not appear in the Acknowledgments section or other areas of your manuscript. We will only publish funding information present in the Funding Statement section of the online submission form.

Please remove any funding-related text from the manuscript and let us know how you would like to update your Funding Statement. Currently, your Funding Statement reads as follows:

“This study was funded by the Bill and Melinda Gates Foundation (OPP1182519). The authors of this paper have no conflicts of interest to report. The grant was warded to RR as the principle investigator.

<https://www.gatesfoundation.org/>

The funders had no role in study design, data collection and analysis, decision to publish, or preparation of the manuscript.”

Please include your amended statements within your cover letter; we will change the online submission form on your behalf.

**Thank you for this explanation. We have now removed the acknowledgments section, as no further acknowledgements are needed. We also approve the Funding Statement drafted by your editorial team above--there are no further edits to the Funding Statement.**

5. Thank you for stating the following in your Competing Interests section:

“No authors have competing interests”

Please complete your Competing Interests on the online submission form to state any Competing Interests. If you have no competing interests, please state ""The authors have declared that no competing interests exist."", as detailed online in our guide for authors at <http://journals.plos.org/plosone/s/submit-now>

 This information should be included in your cover letter; we will change the online submission form on your behalf.

**Thank you, again, for this explanation. We have now included this Competing Interest statement at the end of our cover letter. We appreciate your willingness to edit the online submission form on our behalf.**

6. Please include your full ethics statement in the ‘Methods’ section of your manuscript file. In your statement, please include the full name of the IRB or ethics committee who approved or waived your study, as well as whether or not you obtained informed written or verbal consent. If consent was waived for your study, please include this information in your statement as well.

**Thank you for this note. We have now reviewed the ethics statement presented in the second paragraph of the Methods section to ensure that it contains all required information. We have also revealed the name of the IRB that approved the study, as this was masked in the previous version for blind review.**

**Reviewers' comments:**
1. Is the manuscript technically sound, and do the data support the conclusions?
The manuscript must describe a technically sound piece of scientific research with data that supports the conclusions. Experiments must have been conducted rigorously, with appropriate controls, replication, and sample sizes. The conclusions must be drawn appropriately based on the data presented.

Reviewer #1: Yes

Reviewer #2: Yes

**Thank you**

2. Has the statistical analysis been performed appropriately and rigorously?

Reviewer #1: Yes

Reviewer #2: Yes

**Thank you**

3. Have the authors made all data underlying the findings in their manuscript fully available?

The [PLOS Data policy](http://www.plosone.org/static/policies.action#sharing) requires authors to make all data underlying the findings described in their manuscript fully available without restriction, with rare exception (please refer to the Data Availability Statement in the manuscript PDF file). The data should be provided as part of the manuscript or its supporting information, or deposited to a public repository. For example, in addition to summary statistics, the data points behind means, medians and variance measures should be available. If there are restrictions on publicly sharing data—e.g. participant privacy or use of data from a third party—those must be specified.

Reviewer #1: Yes

Reviewer #2: Yes

**Thank you**

4. Is the manuscript presented in an intelligible fashion and written in standard English?

PLOS ONE does not copyedit accepted manuscripts, so the language in submitted articles must be clear, correct, and unambiguous. Any typographical or grammatical errors should be corrected at revision, so please note any specific errors here.

Reviewer #1: Yes

Reviewer #2: Yes

**Thank you**

5. Review Comments to the Author

Reviewer #1: This is a well written manuscript and the statistical analyses approach in a stepwise fashion is routine for this type of question. The sample size is certainly adequate and the power needed to address the hypotheses may be adequate, although the investigators are counting on the original randomized trial to accommodate this concern. Admittedly the study is cross sectional.

The limitations are well explained and the conclusions follow from the analyses provided. The investigators used hierarchical linear equations to analyze interactions between predictors at various levels and efficacy to predict behavioral intention. The moderators incorporated into the model are certainly reasonable. There is no mention of any fit statistics examining the appropriateness of the linear models unless this is already discussed in previous work and should be referenced if such is the case.

**Thank you for your thoughtful review and summary of the strengths and limitations of our study. We report the adjusted R-square as the fit statistic for the model with the main effects, as well as changes in adjusted R-square with the addition of each moderator (page 15-16). We have now edited the manuscript so that this clearer.**

Reviewer #2: This paper resorts the concept of efficacy to bring together normative factors at the social-level, specifically including descriptive norms, injunctive norms and collective norms, and risk perception at the individual-level, and analyzes data from the RANI project through hierarchical linear equations, and then examines the role of a series of predictors and efficacy on the intention to consume IFA. The main findings are the strength of the association between efficacy beliefs and intentions and the interaction effect involving efficacy beliefs.
The literature review of this paper is well prepared, the hypotheses are natural and reasonable, and the conclusions are generally credible. It is an honor to read this paper, but there are still a few suggestions before publication:
1. It is suggested that the authors supplement the hierarchical linear equations used in this paper at appropriate place, and further enrich the summary statistics on measures;

**Thank you for your thoughtful and careful review of our manuscript. We have not included hierarchical linear equations in our manuscript because we only present models with variables measured at a singular level (the individual level). Rather, the “hierarchical regressions” referred to in this paper is in reference to the step-wise fashion in which the interaction terms were included into the model.**

2. It is suggested that the authors provide further explanations for “low reliability” and “exponentially iterative process” in Measures.

**Thank you for this note. We have edited the manuscript to reflect that we are referring to the low Cronbach’s alpha when we use claim the measure has low reliability, and that the iterative process used was Tukey’s ladder of powers.**

3. As the authors mention the relationship between efficacy and behaviors in Limitations, it is also recommended that they explore the self-efficacy and response efficacy separately

**Thank you for this note. We have looked at efficacy as separate variables and have not found a difference in findings between the two. However, after much consideration, we have decided to report efficacy as a combination of self-efficacy and response-efficacy because the RPA framework indicates that it is the combination between the two that has the power to strengthen or attenuate the influence of risk perception on behavior intentions.**

4. The dependent variable in this paper is the intention to consume IFA, not the actuals. If permitted, it is recommended that the authors continue to track the behavioral performance of participants to better supplement and evaluate the conclusions involved in this paper.

**We appreciate this comment and agree that behavior is important to track. We are delighted to share that the behavioral impact of the RANI Project will be reported in the intervention’s final impact paper. However, in terms of this particular paper, we have chosen to look at intention rather than behavior because 1) we wanted to stay true to the theories used; the TNSB and the RPA framework posit that risk perception, efficacy, and social norms predict intentions, which then subsequently impact behavior and 2) intention and behaviors are measured 6 to 12 months a part in subsequent rounds of the RANI Project’s data collection, therefore we do not report associations between intentions and behavior here due to the temporal distance between the two measures. However, we have added a statement under our description of the measurement of intentions that supports the association between intentions and behaviors.**
